# Mode of Parainfluenza Virus Transmission Determines the Dynamics of Primary Infection and Protection from Reinfection

**DOI:** 10.1371/journal.ppat.1003786

**Published:** 2013-11-21

**Authors:** Crystal W. Burke, Olga Bridges, Sherri Brown, Richard Rahija, Charles J. Russell

**Affiliations:** 1 Department of Infectious Diseases, St. Jude Children's Research Hospital, Memphis, Tennessee, United States of America; 2 The Animal Resource Center, St. Jude Children's Research Hospital, Memphis, Tennessee, United States of America; 3 Department of Microbiology, Immunology and Biochemistry, University of Tennessee Health Science Center, Memphis, Tennessee, United States of America; National Institutes of Health, United States of America

## Abstract

Little is known about how the mode of respiratory virus transmission determines the dynamics of primary infection and protection from reinfection. Using non-invasive imaging of murine parainfluenza virus 1 (Sendai virus) in living mice, we determined the frequency, timing, dynamics, and virulence of primary infection after contact and airborne transmission, as well as the tropism and magnitude of reinfection after subsequent challenge. Contact transmission of Sendai virus was 100% efficient, phenotypically uniform, initiated and grew to robust levels in the upper respiratory tract (URT), later spread to the lungs, grew to a lower level in the lungs than the URT, and protected from reinfection completely in the URT yet only partially in the lungs. Airborne transmission through 7.6-cm and 15.2-cm separations between donor and recipient mice was 86%–100% efficient. The dynamics of primary infection after airborne transmission varied between individual mice and included the following categories: (a) non-productive transmission, (b) tracheal dominant, (c) tracheal initiated yet respiratory disseminated, and (d) nasopharyngeal initiated yet respiratory disseminated. Any previous exposure to Sendai virus infection protected from mortality and severe morbidity after lethal challenge. Furthermore, a higher level of primary infection in a given respiratory tissue (nasopharynx, trachea, or lungs) was inversely correlated with the level of reinfection in that same tissue. Overall, the mode of transmission determined the dynamics and tropism of primary infection, which in turn governed the level of seroconversion and protection from reinfection. These data are the first description of the dynamics of respiratory virus infection and protection from reinfection throughout the respiratory tracts of living animals after airborne transmission. This work provides a basis for understanding parainfluenza virus transmission and protective immunity and for developing novel vaccines and non-pharmaceutical interventions.

## Author Summary

Parainfluenza viruses are highly contagious, a leading cause of acute respiratory infection (ARI) in children, often reoccurring, and currently controlled by non-pharmaceutical interventions. We tracked infection and reinfection of a prototypic murine parainfluenza virus after contact or airborne transmission. Our studies reveal that the mode of transmission determines the dynamics of primary infection. Additionally, higher levels of protection from reinfection are induced in individual respiratory tissues by higher levels of primary infection in those same tissues. Natural infection after either contact or airborne transmission tends to initiate in the URT, but not the lungs. Complete protection from infection in the URT was afforded by URT-biased, non-pathogenic infection after low-dose intranasal vaccination. Overall, the data suggest that parainfluenza virus transmission may be effectively controlled by handwashing, disinfection of surfaces, and environmental control of short-range transmission, in addition to the development of live attenuated vaccines that target the URT.

## Introduction

The *Paramyxoviridae* family includes a number of important human pathogens that are transmitted by the respiratory route including the human parainfluenza viruses (HPIVs), human respiratory syncytial virus (HRSV), human metapneumovirus (HMPV), measles virus, and mumps virus [1]. HRSV, HMPV, and the HPIVs are leading causes of acute respiratory tract infections (ARIs) and pediatric hospitalizations, yet there are no licensed vaccines available for these non-segmented, negative strand RNA viruses [2]. With respect to parainfluenza virus (PIV) infection, the focus of this study, approximately 80% of children are seropositive against HPIV1, HPIV2, and HPIV3 by age 5 [3]. The HPIVs cause of spectrum of respiratory diseases in children including rhinorrhea, cough, laryngotracheobronchitis (croup), bronchiolitis, and pneumonia [4], [5]. The majority of cases are mild upper respiratory tract (URT) infections [6]. However, the HPIVs are a leading cause of ARI hospitalization in children under 5 with HPIV1, HPIV2, and HPIV3 causing an estimated 28,900, 15,600, and 52,000 hospitalizations annually in the United States, a burden typically higher than influenza virus [7]. In the immunocompromised host, HPIV infection can be persistent [8]–[10] and cause severe lower respiratory tract disease that often leads to death [11], [12]. Currently no licensed antiviral therapeutics or immunizations are available against the PIVs. Considering these facts, an understanding of the dynamics of PIV transmission and how the mode of transmission contributes to pathogenesis and protection from reinfection would greatly aid in efforts to control pediatric infections and associated illness.

Respiratory virus transmission can occur through contact with infectious fluids, either directly or indirectly through contaminated fomites, or through inhalation of airborne particles in the form of large droplets or small droplet nuclei [13]. In early experiments with highly symptomatic HRSV-infected infants, caregivers in contact with infected infants or their environment became infected with HRSV while those nearby, but not in contact, did not develop infection [14]. Furthermore, HRSV was found to survive for up to 6 hours on non-porous surfaces and up to 30 minutes on hands [15]. Based on these experiments, respiratory paramyxoviruses such as HRSV are generally believed spread through contact and large airborne droplet transmission [16]. Few studies have specifically investigated HPIV transmission. Infectious HPIV1 virus was recovered from air samples taken 60 cm away from only 1 of 30 HPIV1-infected children, making transmission by small droplet nuclei unlikely [17]. Similar to HRSV, the HPIVs can be recovered from experimentally contaminated non-porous surfaces for up to 10 hours [18]; however, HPIV-3 quickly lost infectivity when placed on the hands [19]. Beyond these studies, surprisingly little is known about HPIV transmission in humans.

The study of respiratory virus transmission in humans is difficult due to ethical, safety, environmental, and budgetary considerations. For these reasons, the use of small animal models to study transmission of respiratory viruses has been widely utilized. The HPIVs poorly infect mice, and HPIV infection in cotton rats, hamsters, guinea pigs, and ferrets is usually asymptomatic with minimal or undetectable pathology [2]. As a result the murine counterpart of HPIV1, Sendai virus (SeV), has been used as a model to investigate PIV pathogenesis and transmission in its natural host [20]–[22]. SeV and HPIV1 are similar in amino-acid sequence identity [23], tissue tropism, and epidemiology [2], [20]. Both viruses elicit cross-protective immunity [24]–[26]. Early transmission experiments using SeV found that the virus readily transmits when mice are placed in direct contact with each other [27]–[30]. However, transmission was less efficient when animals are separated by as little as 2.5–10.5 cm [28]. The ability of SeV to transmit over longer distances has been unclear as there are conflicting reports on its occurrence in the literature [27], [28].

To our knowledge, no previous transmission studies have measured in individual, living animals the kinetics and dynamics of respiratory virus infection and reinfection throughout the respiratory tract. Previous transmission studies have been limited to either nasal washes in living animals or endpoint experiments in which groups of animals are euthanized at defined times after exposure in order to measure viral loads in the nasal cavity, trachea, and lungs. We have developed a SeV reporter virus, rSeV-luc(M-F*), that maintains a wild-type-like phenotype *in vitro* and *in vivo* and allows for the longitudinal study of SeV infection, transmission, and reinfection in individual, living animals [21]. This previous work revealed a tissue-specific dichotomy in which robust infection in the nasopharynx and trachea (even under conditions of a low inoculated dose, an attenuated virus, or host resistance to lung infection) supports efficient contact transmission while the extent of infection in the lungs and the host response determines disease severity. Here, we used this system to gain insight into respiratory virus transmission, assessing the ability of SeV to transmit by both contact and airborne routes. Our data reveal that the mode of transmission (contact versus airborne) determines the timing of transmission, the dynamics of primary infection in the respiratory tract after transmission, and the tissue-specific magnitude of protection from delayed secondary reinfection.

## Materials and Methods

### Ethics statement

All animal studies were approved by the Animal Care and Use Committee of St. Jude Children's Research Hospital (protocol number 459) and were performed in compliance with relevant institutional policies, the Association for the Accreditation of Laboratory Animal Care guidelines, the National Institutes of Health regulations, and local, state, and federal laws.

### Virus, animals, and inoculations

The rSeV-luc(M-F*) virus has been described [21]. Six to eight week-old female 129X1 mice (Jackson Laboratories) were used in all studies. Mice were anesthetized using isoflurane (Baxter Health Care Corporation) and inoculated intranasally (i.n.) with 70- or 7,000-plaque forming units (PFU) rSeV-luc(M-F*) in 30 µL PBS with Ca^2+^ and Mg^2+^. Animals were monitored daily for weight loss, morbidity, and mortality. Inhaled isoflurane was selected as the method of anesthesia because pilot experiments demonstrated 129X1 mice inoculated with 7,000 PFU of virus under isoflurane anesthesia had an average weight loss of 17.5% and a mortality rate of 10%, whereas avertin-anesthetized mice suffered greater weight loss (24.5%) and mortality (80%).

### Bioluminescence imaging

Prior to imaging, mice were injected intraperitoneally (i.p.) with luciferin (Caliper Corp.) at a dose of 150 mg/kg of body weight and anesthetized with isoflurane for 5 min. *In vivo* images were acquired for 1 min with a binning of 4 using the IVIS CCD camera system. Images were analyzed with Living Image 4.2 software (PerkinElmer). To quantify bioluminescence signal, square regions of interest were drawn around the nasopharynx, trachea, and lungs and the total flux (photons/sec) was measured. In drawing regions of interest, demarcations between respiratory tissues was made using external reference points that were found to correspond to internal anatomy after dissection. The line of demarcation between the nasopharynx and trachea was the base of the jaw and between the trachea and lungs was just above the manubrium where the trachea bifurcates into left and right bronchi. Adjustments in definition of the regions of interest did not substantially alter the infection phenotypes (Figure S1). Bioluminescence curves were graphed over time, and the area under the curve was measured using GraphPad Prism software with zero as the baseline.

### Transmission experiments

**Figure 1:**
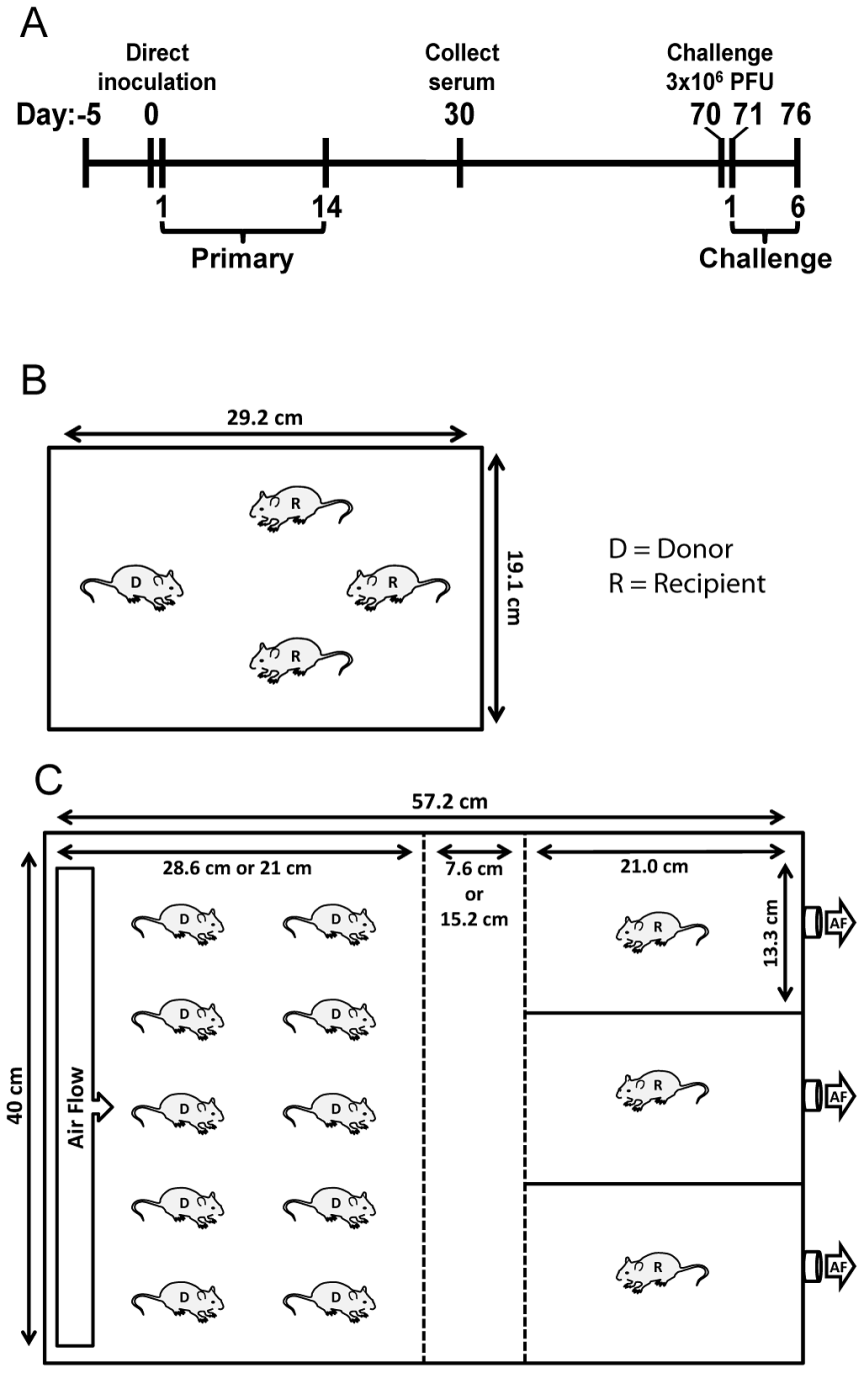
Schematic of experimental and transmission cage design. (**A**) Timeline of experiment. For airborne transmission experiments, mice were placed in cages 5 days prior to start of experiment for acclimation. Bioluminescence and weight loss were monitored daily after direct inoculation of donor mice during primary infection and rSeV-luc(M-F*) challenge on day 70 of the experiment. (**B**) Dimensions and number of mice per cage for contact transmission experiments. (**C**) Dimensions and number of mice per cage for airborne transmission experiments. In initial experiments, the separation between donor and recipient mice was 7.6 cm. For the longer-range airborne experiments, the left middle divider was moved an additional 7.6 cm to the left for a total separation of 15.2 cm. Solid lines denote solid surfaces that do not permit air flow, and dashed lines indicate stainless steel mesh barriers that permit air flow. D = donor animal, R = recipient animal, and AF = air flow.

Airborne transmission cages were designed with assistance from the St. Jude animal husbandry manager and constructed by the St. Jude Biomedical Engineering Department. Dimensions of the cages are (57.2 cm×40 cm) with a stainless steel, movable divider that allowed a void space of either 7.6 or 15.2 cm between the chamber housing the infected donor animals and the naïve recipient animals (Figure 1C). Air entered the cages starting in the donor chamber most distal to recipient animals (left of Figure 1C) through 4 rows of 12 holes that were 3 mm in diameter. The rows were positioned at 3, 4, 10, and 11 cm from the bottom of the cage. Air was pulled through the cages and exited from one hole in each of the recipient mouse isolation chambers (right of Figure 1C). To facilitate air flow from the direction of donor mice to recipients, the 3 holes in isolation chambers were connected to the room air-handling exhaust (5 complete room air changes/hour). The cages were sealed on top with plastic lids instead of filter tops so that the air would flow from donor to recipient animal. The movable dividers between donor mice and recipient isolation chambers were constructed of stainless steel and contained 15 rows of 28 holes that were 1-cm diameter. Food was supplied in glass bowls, and water was made available in gel form (Napa Nectar; System's Engineering). For airborne transmission experiments, ten donor animals were inoculated and placed in the cage with 3 individually isolated naïve animals. For contact transmission, donor animals were inoculated and 24 h later were placed individually into cages containing 3 naïve contact mice. For both airborne and contact transmission experiments, donor mice were inoculated i.n. with 70- or 7,000-PFU rSeV-luc(M-F*). Bioluminescence, weight loss, morbidity, and mortality were monitored daily for 14 days in all of the naïve recipient animals. All donors for contact transmission and 3 of 10 donors for airborne transmission were similarly monitored. Seventy days after the inoculation of donor mice, animals were challenged i.n. with a lethal dose of 3×10^6^-PFU rSeV-luc(M-F*); bioluminescence, weight loss, morbidity, and mortality were measured daily. The time to detection was reported as the first day when bioluminescence signal was ≥5.5 log_10_ photons/sec in any respiratory tissue (nasopharynx, trachea, or lungs). Temperature and relative humidity inside each cage were measured every 30 minutes using a Hobo Data Logger that was placed in the void between donor and recipient mice for duration of transmission experiments.

### ELISA assays

Serum was collected from anesthetized animals 30 days after the inoculation of donor mice, and SeV-specific ELISAs were used to measure the level of SeV-specific antibody present. Briefly, 96-well plates were coated overnight with disrupted purified SeV particles (10 µg/ml). Plates were blocked with PBS containing 3% BSA and then incubated with 10-fold serially diluted serum samples. After incubation, plates were washed, probed with HRP-Goat anti-mouse IgG (Southern Biotechnologies) and then washed further. To quantify levels of SeV-specific antibodies present, TMB substrate buffer was added to the wells followed by stop solution and absorbance was read at a wavelength of 450 nm. GraphPad Prism non-linear regression software was used to calculate antibody titers.

### Statistical analysis

Statistical analyses were performed with Prism software version 5.0 (GraphPad Software). Statistical significance of weight loss was performed using a two-way ANOVA. Samples were analyzed using a two-tailed unpaired Student's *t*-test; for samples with unequal variance a Welch's correction was performed. Correlation coefficients were calculated using linear regression analyses.

## Results

### Dynamics of infection and reinfection in donor mice as a function of inoculated dose

**Figure 2:**
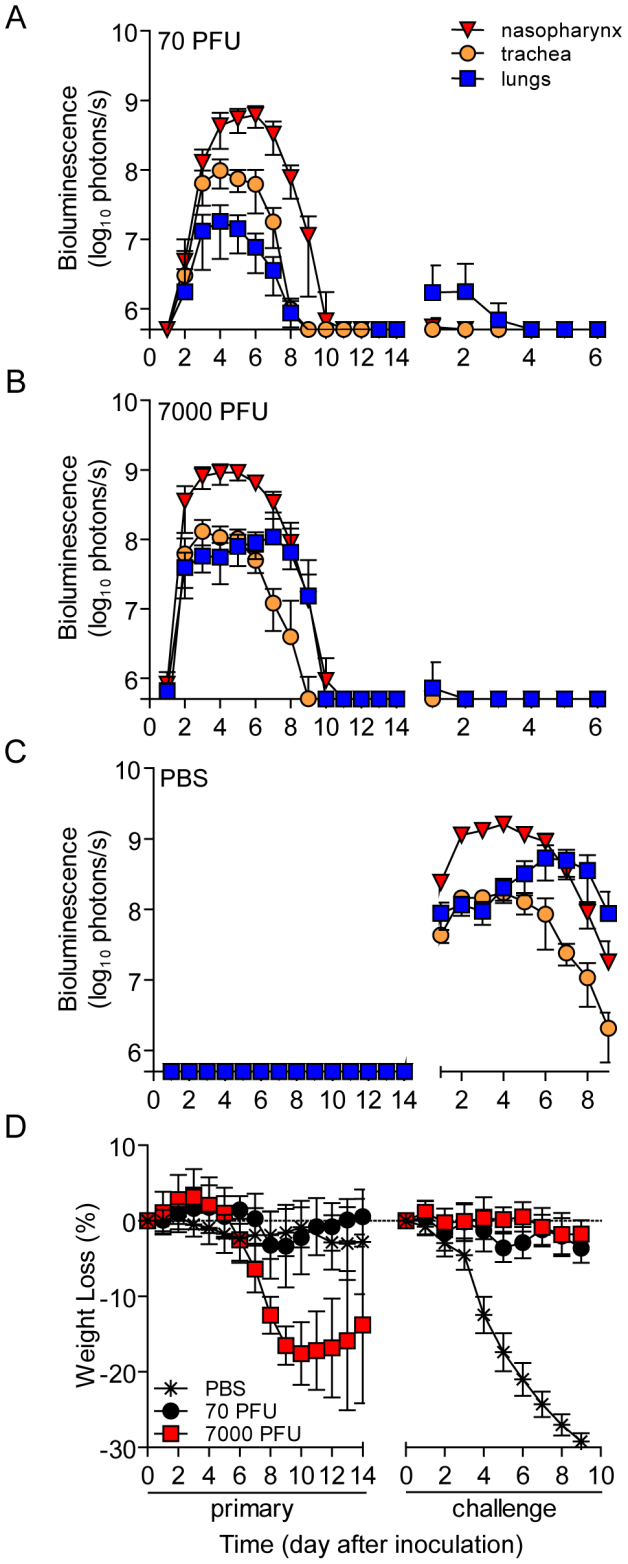
Bioluminescence and weight loss in directly inoculated mice. 129X1 mice were inoculated intranasally with 70- (**A**) or 7,000- (**B**) PFU of rSeV-luc(M-F*), or PBS (**C**) and bioluminescence in the nasopharynx (red triangles), trachea (orange circles), and lungs (blue squares) was measured for 14 days. Seventy days after the initial inoculation, the same mice were challenge with a lethal dose of 3×10^6^-PFU of rSeV-luc(M-F*) and bioluminescence was again measured daily. (**D**) Weight loss was used as a measure of morbidity and was monitored throughout the course of experiment. All numbers are reported as the means ± the standard deviation (70-PFU *n* = 22 and 7,000-PFU *n* = 48). The bottom of the y-axis is 5.5×10^5^ photons/s, the limit of detection of bioluminescence.

To investigate how the mode of transmission influences primary infection and protection from reinfection in living mice, we used non-invasive bioluminescence imaging. Using the wild-type-like virus rSeV-luc(M-F*), which expresses the firefly luciferase reporter gene in infected cells, we previously demonstrated that the magnitude of bioluminescence in intact mice correlates with *ex vivo* tissue titers of infectious virus in the nasopharynx, trachea, and lungs [21]. This previous work also shows wild-type SeV and rSeV-luc(M-F*) have similar replication kinetics in LLC-MK2 cells and in the nasal turbinates, trachea, and lungs of infected mice; both viruses also induced similar levels of weight loss and mortality, lymphocyte infiltration in bronchoalveolar lavage fluid, and SeV-specific antibody titers. To establish a transmission model, we first investigated the dynamics of primary infection and protection from lethal challenge in donor 129X1 mice that had been inoculated intranasally with either a relatively low (70-PFU) or high (7,000-PFU) dose of rSeV-luc(M-F*) and were subsequently challenged with a lethal dose of 3×10^6^-PFU rSeV-luc(M-F*) 70 days later (Figure 1A). Direct intranasal inoculation of donor mice with a 70-PFU dose of virus resulted in a robust primary infection in the nasopharynx and trachea but limited infection in the lungs (Figure 2A), consistent with the 70-PFU inoculated mice having no weight loss compared to uninfected animals (Figure 2D). While donor mice directly inoculated with the higher 7,000-PFU dose had similarly high levels of primary infection in the nasopharynx and trachea, the higher dose resulted in 10-fold greater infection in the lungs, delayed clearance in the lungs, and an average weight loss of approximately 20% (Figures 2B,D).

PBS-inoculated animals challenged with 3×10^6^-PFU rSeV-luc(M-F*) lost up to 30% starting weight (the limit in our protocol) and had a mortality rate of 100% (10/10 mice). These mice also displayed high peak levels of bioluminescence throughout the respiratory tract (1.61×10^9^, 1.71×10^8^, and 5.32×10^8^ photons/s in the nasopharynx, trachea, and lungs, respectively) (Figure 2C). These maxima correspond to tissues titers of 1.43×10^9^, 1.7×10^8^, and 4.97×10^8^ PFU/mL in the nasopharynx, trachea, and lungs, respectively, using a previously determined titration [21]. 70 days after primary inoculation, both 70- and 7,000-PFU donor groups were protected from intranasal challenge with 3×10^6^-PFU rSeV-luc(M-F*), suffering no significant weight loss (Figure 2D) and no mortality. Both groups did not display bioluminescence in the nasopharynx or trachea after day-70 challenge, and both groups had low levels of bioluminescence in the lungs (<10^7^ photons/s) that was cleared after 3 or 2 days for the 70- and 7,000-PFU groups, respectively. Overall, the data showed that primary infection in the lungs of the 7,000-PFU inoculated group was 10-fold greater and was cleared later than in the 70-PFU group, yet the peak levels of primary infection in the nasopharynx and trachea and the level of protection from lethal secondary challenge were similar in both groups.

### URT-dominant primary infection after contact transmission protects from lethal challenge

**Figure 3:**
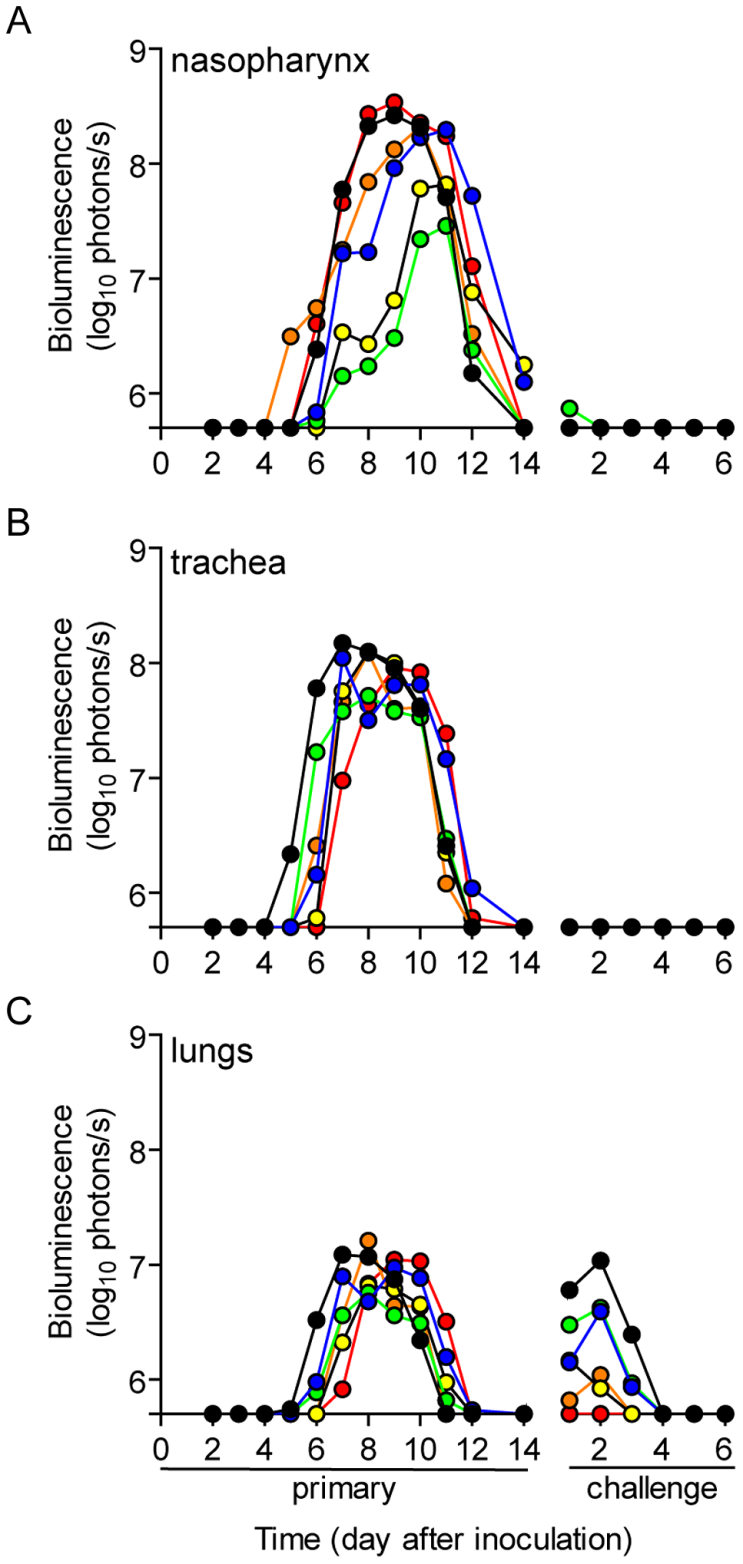
Dynamics of infection after contact transmission and subsequent challenge. One 129X1 mouse inoculated intranasally with 70-PFU of rSeV-luc(M-F*) was placed in a cage with 3 naïve mice 24 hours later. Bioluminescence in the (**A**) nasopharynx, (**B**) trachea, and (**C**) lungs was measured daily in the contact mice until infection was cleared (on average 14 days). Seventy days after inoculation of donor mice, the recipients were challenged with 3×10^6^ PFU of rSeV-luc(M-F*) so that reinfection could be monitored by bioluminescence. Each individual mouse is color-coded (*n* = 6). The bottom of the y-axis is 5.5×10^5^ photons/s, the limit of detection of bioluminescence.

To investigate the dynamics of infection and extent of protective immunity after contact transmission, we intranasally inoculated donor mice with 70-PFU of rSeV-luc(M-F*) and then placed one donor mouse per cage with 3 naïve recipient mice (Figure 1B). 100% of the animals in contact became infected (Table 1), as assessed by bioluminescence and seroconversion. Primary infection after contact transmission typically initiated within the upper respiratory tract and then spread to the lungs approximately 1 day later (Figure 3). A low level of infection observed in the lungs after contact transmission (peak values of approximately 1×10^7^ photons/s, which corresponds to virus titers of approximately 9.35×10^6^ PFU/mL) was consistent with the animals suffering no weight loss or mortality. The capacity of primary infection after contact transmission to protect from reinfection on day 70 of the experiment was assessed by intranasal challenge of 3×10^6^-PFU of rSeV-luc(M-F*). A lethal challenge dosage is common in vaccination studies and was chosen for these studies in order to better discriminate protection from reinfection in individual respiratory tissues. No reinfection in the nasopharynx and trachea was detected for all 6 recipient mice, yet 5/6 recipient mice displayed a low level of reinfection in the lungs, which was cleared in 4 days or less (Figure 3). Overall, URT-dominant primary infection after contact transmission resulted in complete protection from morbidity and mortality in a lethal challenge model, complete protection from reinfection in the URT, and extensive, albeit in some cases incomplete, protection from reinfection in the lungs.

**Table 1:** Frequency of transmission.

	Direct contact	Airborne transmission^d^		
	transmission^a^	7.6 cm		15.2 cm
Expt. #	70 PFU^b^	70 PFU	7000 PFU	7000 PFU
I	100% (9/9)	67% (2/3)	33% (1/3)	nd
II	nd^c^	100% (3/3)	100% (6/6)	nd
III	nd	100% (3/3)	100% (3/3)	nd
IV	nd	nd	nd	100% (6/6)
V	nd	nd	nd	100% (6/6)
Total	100% (9/9)	89% (8/9)	83% (10/12)	100% (12/12)

a One infected donor animal was introduced into a cage of 3 naïve recipient mice one day after inoculation and housed together until end of experiment. Frequencies are expressed as percentage of transmission (no. positive/total no. naïve animals).

b The contact transmission experiment was completed once using only 70 PFU direct inoculation as previously published data [21] revealed similar frequency of transmission and dynamics of infection after contact transmission regardless of direct inoculation dose.

c nd = not done.

d Individual naïve recipient mice were housed in one of 3 isolation chambers per large transmission cage and were separated from the ten infected donor animals by the indicated distances. 7.6 cm = 3 inches and 15.2 cm = 6 inches. Ten infected animals were placed in transmission cage directly after inoculation for both the 7.6 and 15.2 cm experiments.

### Increased infection in the lungs of donor mice does not enhance short-range airborne transmission

We previously found that increased infection in the lungs of donor mice, due to a higher inoculum, did not increase the frequency or dynamics of infection after contact transmission [21]. We hypothesized that increased virus growth in the lungs of donors would enhance airborne transmission by potentially increasing the number of airborne particles containing virus and/or the number of infectious virions per airborne particle. To test this hypothesis, ten donor mice were inoculated intranasally with either 70- or 7,000-PFU of rSeV-luc(M-F*) and placed in a custom transmission cage that contained three individually isolated recipient mice (Figure 1C). Animal weight and bioluminescence was monitored daily, first in recipient and then in donor mice. The frequency of airborne transmission across the 7.6-cm separation was 89% (8/9) and 83% (10/12) in groups containing 70- and 7,000-PFU inoculated donor mice, respectively (Table 1). Thus, a 10-fold greater and longer-lived SeV infection in the lungs of donor mice, due to a higher-dose inoculation, did not increase the efficiency of airborne transmission. These results suggest that the load of virus in the lungs of donor mice is not the predominant factor governing short-range airborne transmission of Sendai virus.

To confirm that transmission did not occur between the three individually isolated recipient mice in the airborne transmission cages, we performed duplicate lateral transmission experiments using two cages each time. In these experiments, we intranasally inoculated a mouse in the middle chamber with 70 PFU of rSeV-luc(M-F*). None of the eight lateral naïve mice became infected, as evidenced by a lack of bioluminescence and seroconversion.

### Short-range airborne transmission of Sendai virus occurs later than contact transmission

**Figure 4:**
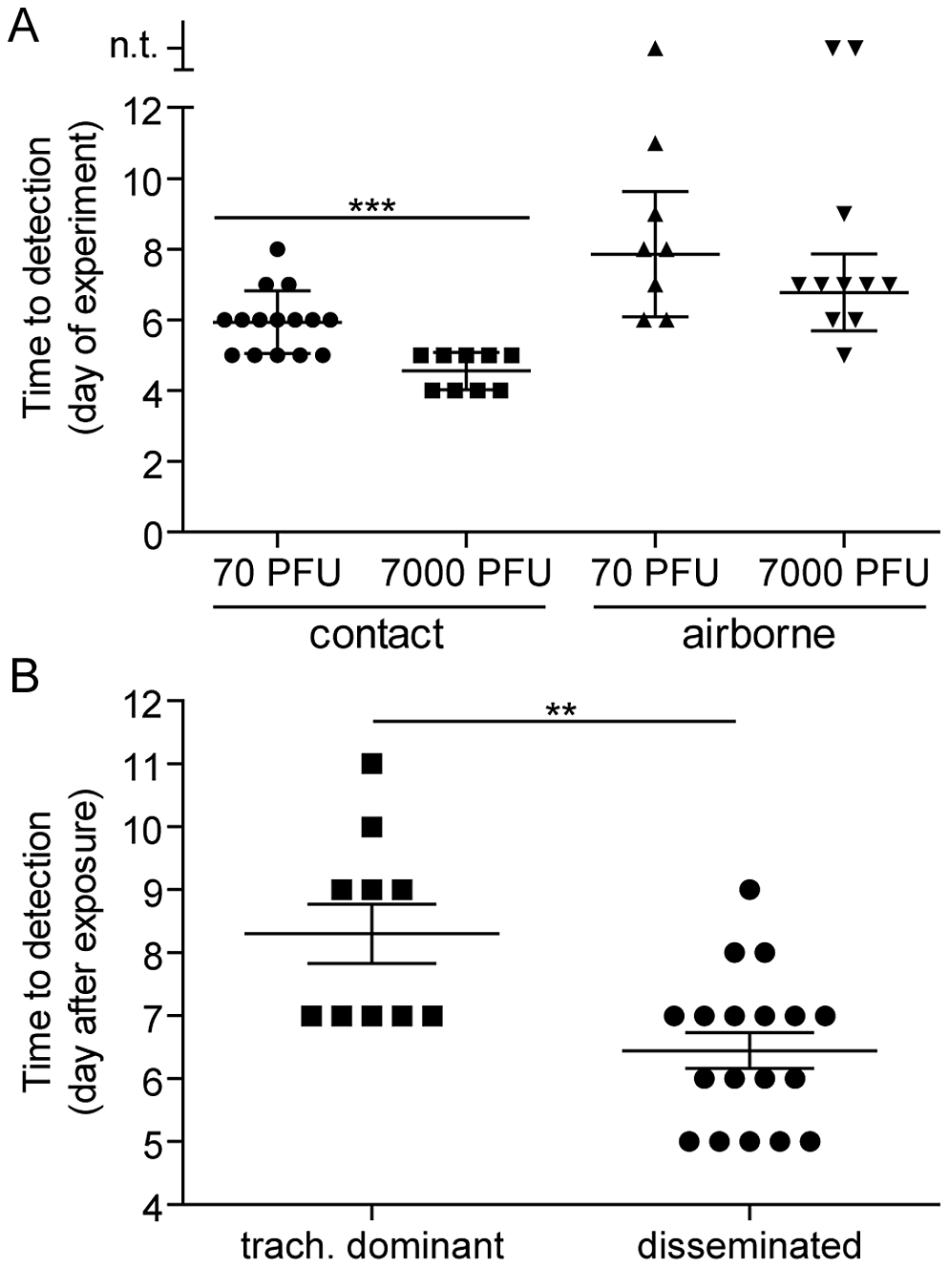
Timing of contact and airborne transmission. (**A**) Transmission time based on the mode of transmission and virus inoculum in donor mice. The day of transmission was recorded based on the day of experiment. Day 0 is the day donor mice were directly inoculated intranasally with either 70- or 7,000-PFU of rSeV-luc(M-F*). For contact transmission, naïve recipient mice were caged with donor mice 1 day after direct inoculation. For airborne transmission, naïve recipient mice were caged with infected donor mice directly after inoculation. Reported is the first day when bioluminescence signal exceeded the limit of detection (5.5 log_10_ photons/sec) in any respiratory tissue. (**B**) Time of airborne transmission based on whether the dynamics of primary infection were tracheal dominant or respiratory tract disseminated. Significance was determined by Student's t-test: *** p = 0.0003 and ** p = 0.001. n.t. on the y-axis of panel A = no transmission.

We previously found that the timing of contact transmission of SeV coincides with high viral loads in the nasal cavity (>10^5^ PFU/mL), both of which occur approximately one day earlier when donor mice are inoculated with 7,000-PFU compared to 70-PFU [21]. In the present study, we measured the timing of transmission by contact and airborne routes. The average time for contact transmission was significantly faster (p = 0.0003) when donor animals were inoculated with 7,000-PFU (4.6 days) versus 70-PFU (5.9 days) (Figure 4A). Short-range airborne transmission occurred on the average two days later than contact transmission for a given inoculated dose in donors. Short-range airborne transmission, when it occurred, was detected approximately one day earlier on the average in groups containing 7,000-PFU inoculated donors (6.8 days) compared to groups with 70-PFU inoculated donors (7.9 days). However, the difference was not statistically significant (p = 0.15) and transmission more frequently occurred in the 70-PFU group than the 7,000-PFU group (89% efficient versus 83%, respectively). Prolonged virus shedding by donors in 7,000-PFU inoculated groups could result in later transmission to newly exposed naïve recipients as late as nine days after inoculation of donors. It is possible that the delay in transmission timing by an airborne route results from the need of a threshold amount of virus to be present in the mucus. Alternatively, virus- or immune cell-mediated epithelial cell damage and sloughing may be necessary to generate the particle sizes necessary to stably transmit virus through the air. To summarize, contact transmission of SeV occurred on average sooner than short-range airborne transmission, and a higher and longer-lived infection in the lungs of donor animals (due to a 100-fold greater inoculated dose) did not increase the frequency or timing of short-range airborne transmission.

### Diverse dynamics of primary infection after short-range airborne transmission

**Figure 5:**
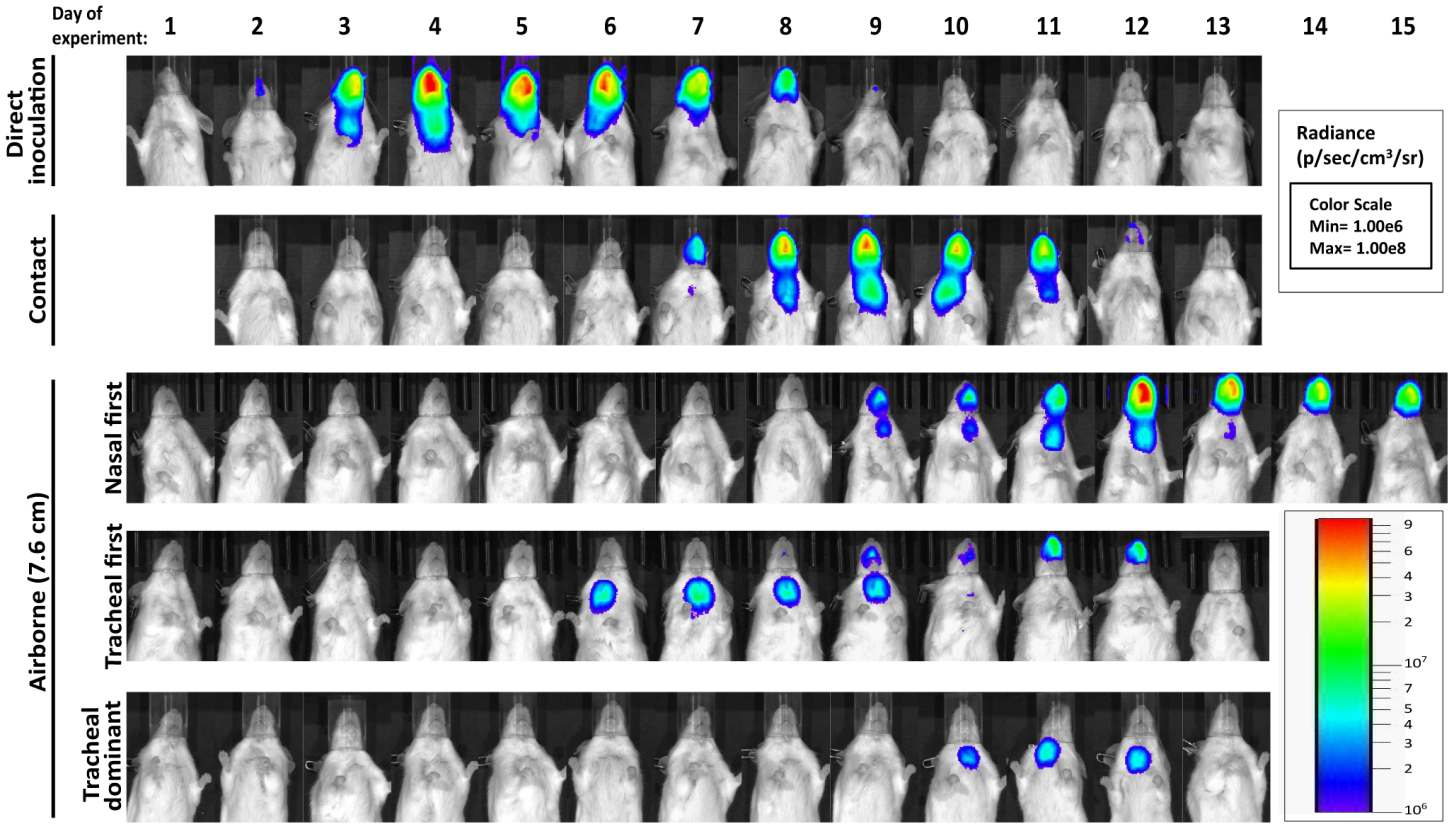
Dynamics of Sendai virus infection for representative, individual mice. Every 24: directly inoculated intranasally with 70-PFU rSeV-luc(M-F*), a contact mouse exposed to a 70-PFU directly inoculated mouse, an airborne-exposed mouse initially infected in the nasopharynx, an airborne-exposed mouse initially infected in the trachea, and an airborne-exposed mouse predominantly infected in the trachea. The data are displayed as radiance (bioluminescence intensity) on a rainbow log scale with a range of 1×10^6^ (blue) to 1×10^8^ (red) photons/s/cm^2^/steradian (inset).

**Figure 6:**
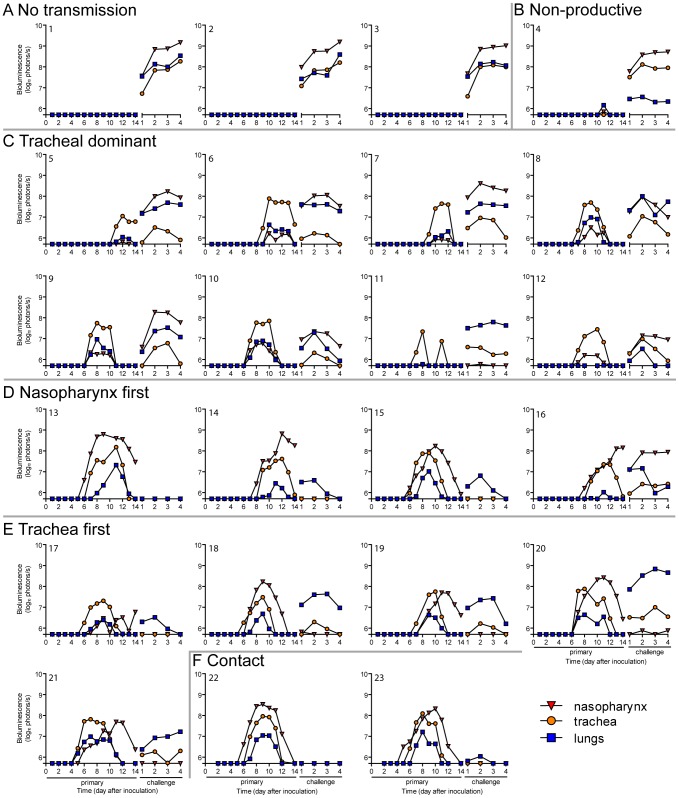
Primary infection and secondary reinfection in individual mice after airborne transmission across a 7.6-cm separation. *In vivo* bioluminescence was measured in individual animals after airborne exposure to donor mice that had been directly inoculated with 70 or 7,000 PFU of rSeV-luc(M-F*). All mice were challenged on day 70 of the experiment with a lethal 3×10^6^-PFU dose of rSeV-luc(M-F*). Mice involved in the airborne transmission experiments were categorized based on the dynamics of the resultant infection: (**A**) no transmission (1–3), (**B**) non-productive infection (4), (**C**) tracheal dominant (5–12), (**D**) respiratory disseminated with nasopharyngeal first (13–16), and (**E**) respiratory disseminated with tracheal first (17–21). (**F**) Bioluminescence curves for two representative mice infected by contact transmission are also included. The bottom of the y-axis is 5.5×10^5^ photons/s, the limit of detection of bioluminescence.

**Figure 7:**
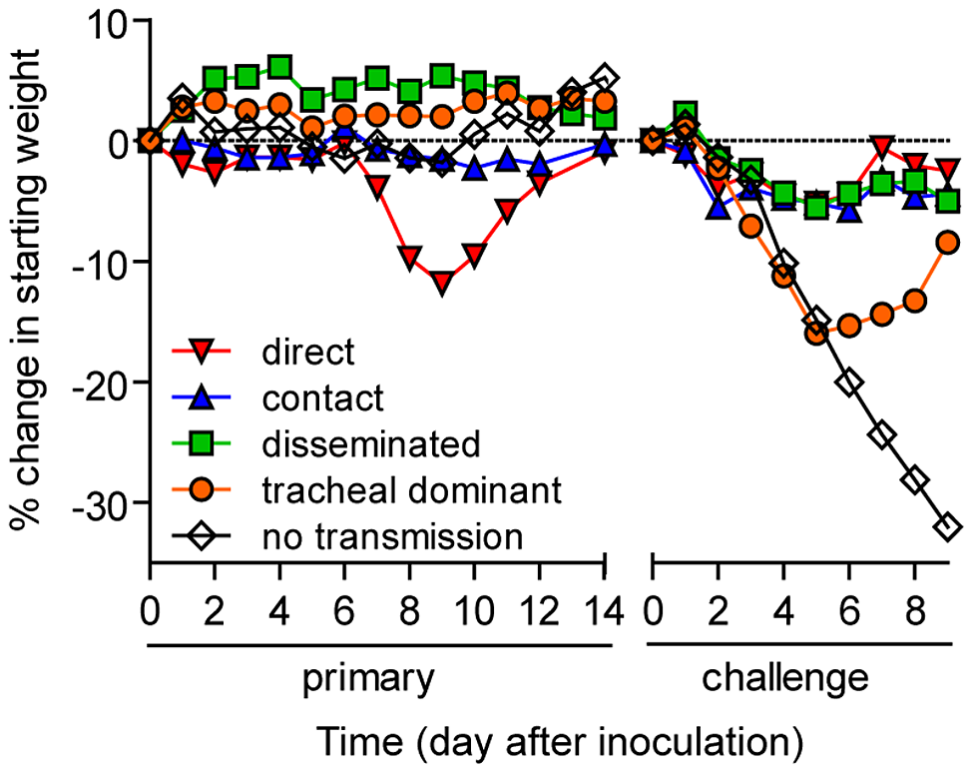
Mean percent weight change during the airborne transmission experiment with a 7.6-cm separation. For airborne transmission experiments mice were categorized based on the dynamics of infection after transmission. Duplicate contact and triplicate airborne experiments were performed.

A major gap in the field of respiratory virus transmission is an understanding of the dynamics of primary infection throughout the respiratory tract after airborne transmission. While primary infection after contact transmission initiated in the nasopharynx (Figure 3), we hypothesized that primary infection after airborne transmission would initiate in the trachea and/or lungs due to the inhalation of aerosolized virus-containing particles. Moreover, we expected infection initiating in the trachea or lungs would lead to more severe disease than infection initiating in the nasopharynx. For airborne transmission across a 7.6-cm separation, we found that the timing and magnitude of infection in the nasopharynx, trachea, and lungs varied substantially between individual mice (Figures 5,6). The inoculated dose in donor animals (70- versus 7,000-PFU) appeared to have little, if any, influence on the dynamics of infection after short-range airborne transmission (Table 2). Based on the individual bioluminescence curves (Figure 6), we classified the phenotypes into four categories (Table 2). In one mouse (5%) seroconversion was detected after only a low level of pulmonary bioluminescence on day 11 (non-productive infection; Figure 6B). In 9 mice (43%) infection was disseminated throughout the respiratory tract (Figure 6D,E), initiating first in the nasopharyngeal cavities of 4 mice (19%) and in the trachea of the remaining 5 mice (24%). The “nasopharynx first” and “trachea first” phenotypes will collectively be referred to as respiratory disseminated infections throughout the manuscript despite differences in the initial location of infection. In 8 mice (38%) infection was dominant in the trachea and low levels or no infection was detected in the nasopharynx or lungs. Tracheal-dominant infection was typically cleared in 4 days, whereas it typically took 7 days to clear infection that disseminated throughout the respiratory tract (Figure 6). After short-range airborne transmission, none of the mice had large levels of infection in the lungs and none displayed weight loss or mortality (Figures 6,7). In summary, bioluminescence imaging in intact mice revealed that short-range airborne transmission results in multiple unique phenotypes of primary SeV infection, several of which were more prominent in the trachea than in the nasopharynx and lungs.

**Table 2:** Phenotypes of primary infection after airborne transmission.

	# of mice (%) infected^b^			
	7.6 cm			15.2 cm
Category^a^	70 PFU	7000 PFU	Total	7000 PFU
No transmission	1 (11%)	2 (17%)	3 (14%)	0 (0%)
Respiratory tract disseminated	4 (44%)	5 (42%)	9 (43%)	9 (75%)
Nasopharyngeal first	3 (33%)	1 (8%)	4 (19%)	6 (50%)
Tracheal first	1 (11%)	4 (33%)	5 (24%)	3 (25%)
Tracheal dominant	3 (33%)	5 (42%)	8 (38%)	2 (17%)
Non-productive^c^	1 (11%)	0 (0%)	1 (5%)	1 (8%)
Total (*n*)	9^c^ (100%)	12 (100%)	21 (100%)	12 (100%)

a Phenotypes were categorized selected by comparing the trends of bioluminescent signal within individual animals in each of the following tissues: nasopharynx, trachea, and lungs.

b Mice reported were infected through airborne exposure to donor animals inoculated with either 70 or 7000 plaque forming units of SeV.

c In two mice, bioluminescence was detected at very low levels late after co-housing and the mice seroconverted on day 30. Such an infection is characteristic of non-productive transmission.

### Dynamics of primary infection influences the tropism and magnitude of reinfection

Given the diversity in phenotypes of primary infection after airborne transmission in individual animals, we hypothesized that increased infection in a given respiratory tissue would confer better protection from reinfection in that same tissue. To assess this, we performed a lethal challenge on day 70 of the experiment and monitored reinfection in individual animals using bioluminescence imaging (Figure 6). Day 70 challenge of the no transmission category resulted in high levels of infection throughout the respiratory tract (Figure 6A), up to 30% weight loss (Figure 7), and 100% mortality within 9 days. All mice that had been previously infected after airborne exposure survived challenge. The mouse with non-productive infection was reinfected to a high level (>10^8^ photons/s) in the nasopharynx and trachea, was moderately protected from reinfection in the lungs (<10^7^ photons/s), lost 14% of its body weight, and recovered from the challenge (Figure 6B). Mice that had a tracheal dominant primary infection suffered significant weight loss (Figure 7; p<0.001) and were protected from day 70-challenge to a greater extent in the trachea (typically <10^7^ photons/s) than in the nasopharynx and lungs (usually >10^8^ and >10^7^ photons/s, respectively) (Figure 6C). Mice that had a respiratory tract disseminated infection, whether nasal or tracheal first, were protected from weight loss after day 70 challenge (Figure 7) and typically had lower levels of reinfection in the nasopharynx and lungs (Figure 6D,E) than did mice in the tracheal dominant category.

**Figure 8:**
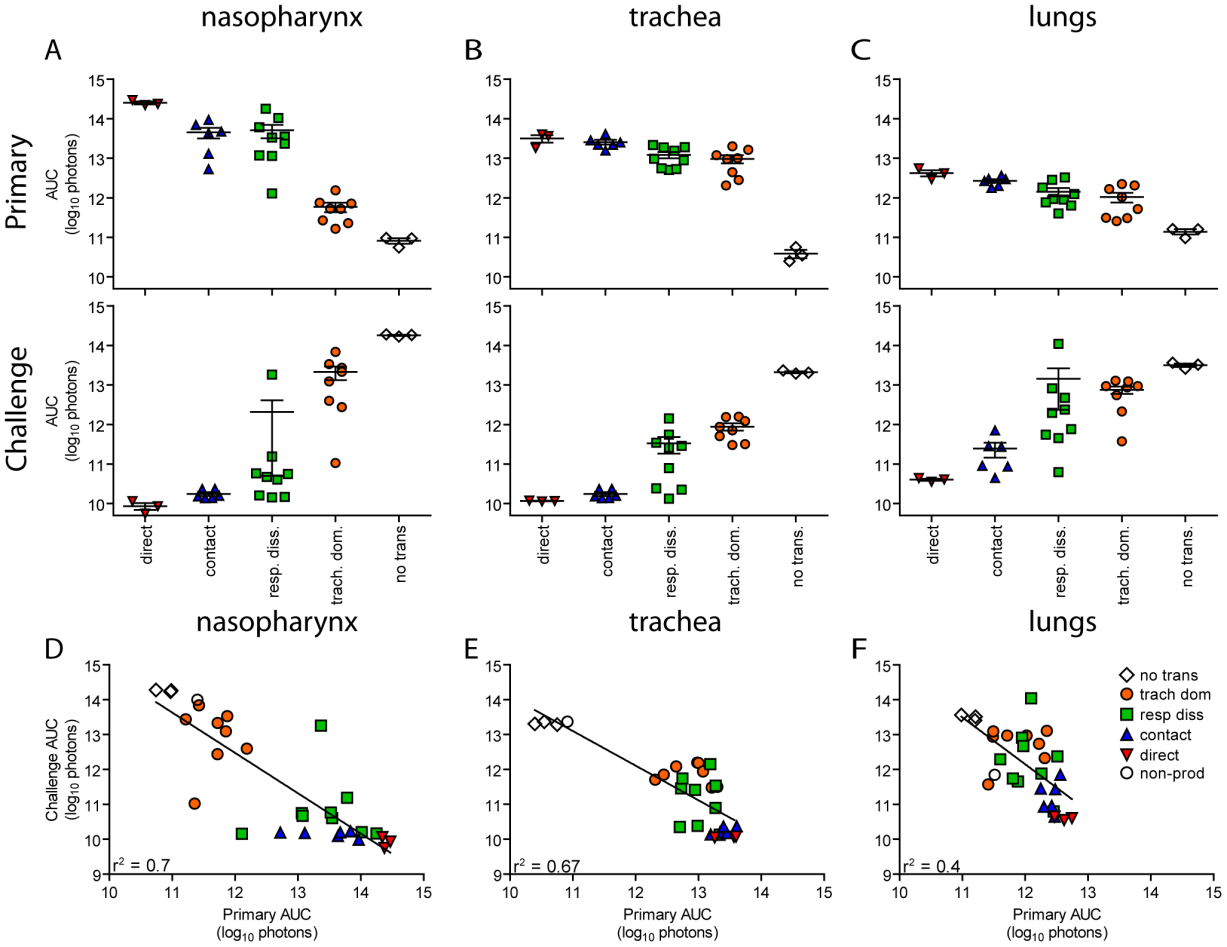
Tissue-specific magnitude of Sendai virus infection in the respiratory tracts of living mice after direct inoculation and transmission. (**A–C**) Overall magnitude of infection of primary and challenge infections as determined by integration of daily measurements of total flux with respect to time. The areas under the curve (AUC) of bioluminescence are expressed as the total amount of photons on a log_10_ scale. The association between the magnitude of primary and challenge infection (AUC) in the nasopharynx (**D**), trachea (**E**), and lungs (**F**) was determined using linear regression analysis (r^2^) with GraphPad Prism software. Data for airborne transmission corresponds to experiments that had a 7.6-cm separation between donor and recipient mice. No trans. = no transmission, trach. dom. = tracheal dominant infection, rep. diss. = respiratory tract disseminated infection.

**Figure 9:**
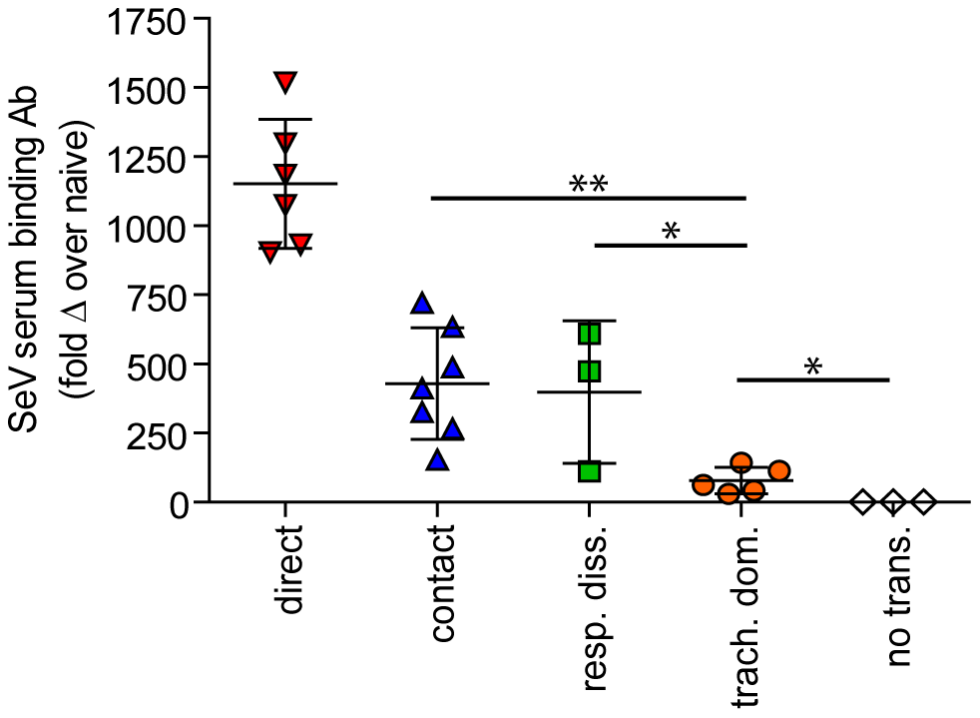
Sendai virus-specific binding antibody titers. Sera were collected on day 30 of the experiment. Titers were measured by reciprocal endpoint dilutions in ELISA assays and the fold change in titers over mock inoculated mouse levels was calculated. Data for airborne transmission corresponds to experiments that had a 7.6-cm separation between donor and recipient mice. Significance was determined using the Student's t-test: * p≤0.03 and ** p = 0.004. No trans. = no transmission, trach. dom. = tracheal dominant infection, rep. diss. = respiratory tract disseminated infection.

In contrast to short-range airborne transmission, mice in the contact transmission group displayed little to no pulmonary reinfection when challenged on day 70 and had no detectable reinfection in the nasopharynx and trachea (Figure 6F). For both contact and airborne transmission, a trend was observed in which a larger amount of primary infection in a given respiratory tissue (nasopharynx, trachea, or lungs) correlated with a greater degree of protection from reinfection. To explore the relationship between primary infection and reinfection, for each individual we calculated the bioluminescence areas under the curve (AUC) for each respiratory tissue (Figure 8) and measured the levels of binding antibodies in peripheral blood sera animal (Figure 9). In general, mice with higher levels of primary infection had higher levels of anti-SeV serum antibody levels and greater levels of protection from reinfection during challenge with the following rank order: directly inoculated>contact transmitted>airborne disseminated>airborne tracheal dominant>no transmission. Mice in the tracheal-dominant category had relatively high levels of infection in the trachea (Figure 8B) but only a low level of serum binding antibodies (Figure 9), presumably due to a low level of infection in the nasopharynx and/or lungs (Figure 8A,C). In summary, the mode of transmission of SeV was found to determine the dynamics of primary infection in mice. The magnitude of primary infection correlated with the extent of protection from reinfection in a given respiratory tissue.

### Airborne transmission over a longer distance and with fewer donor animals

**Figure 10:**
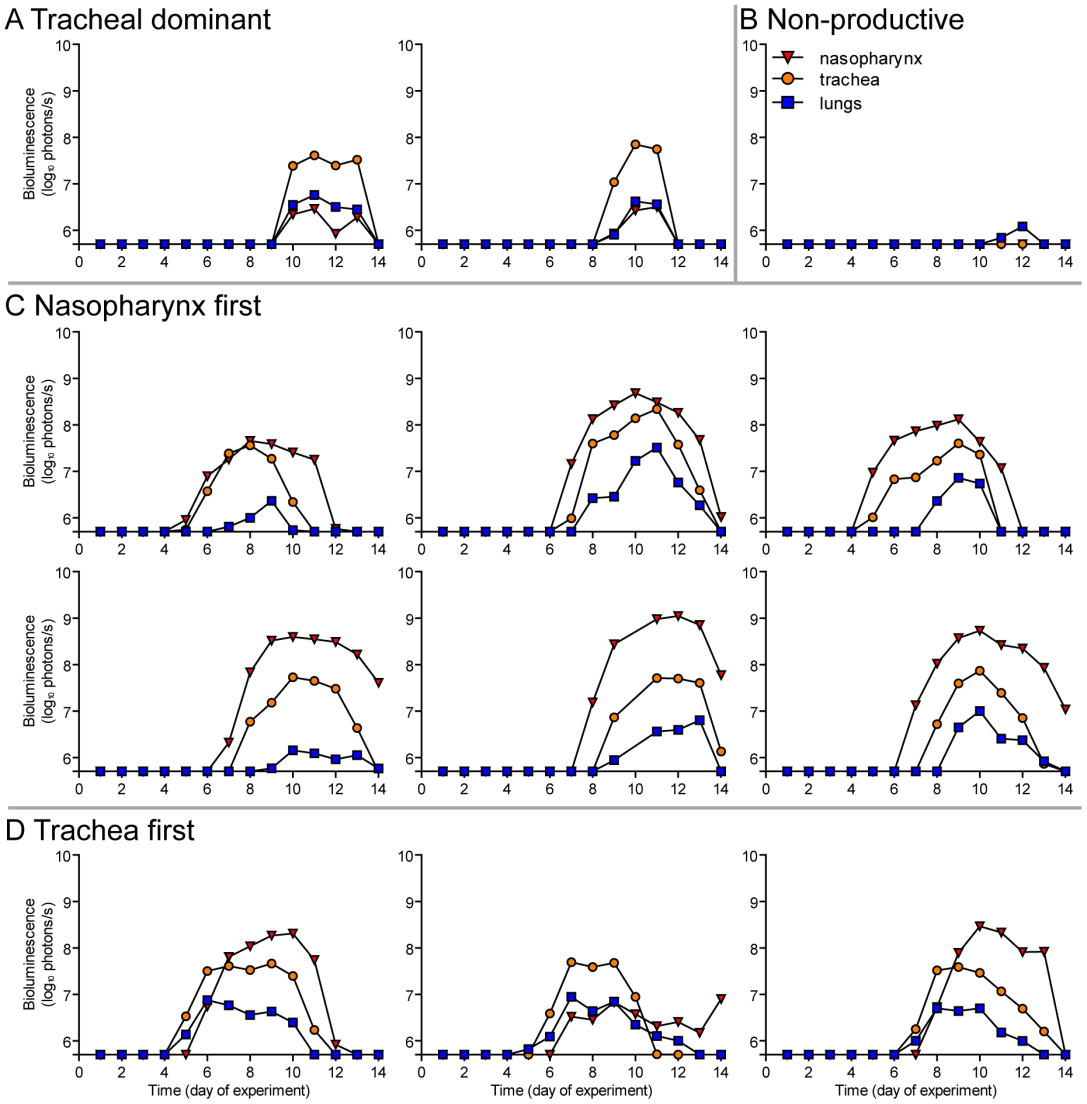
Primary infection in individual mice after airborne transmission across a 15.2-cm separation. *In vivo* bioluminescence was measured in individual animals after airborne exposure to donor mice that had been directly inoculated with 7,000 PFU of rSeV-luc(M-F*). Mice involved in the airborne transmission experiments were categorized based on the dynamics of the resultant infection: (**A**) tracheal dominant, (**B**) non-productive infection, (**C**) respiratory disseminated with nasopharyngeal first, and (**D**) respiratory disseminated with tracheal first. The bottom of the y-axis is 5.5×10^5^ photons/s, the limit of detection of bioluminescence.

Having observed transmission across a 7.6-cm separation, we next investigated if airborne transmission across a separation twice as long would alter the dynamics of primary infection. In each cage, we directly inoculated ten donor mice with 7,000 PFU of rSeV-luc(M-F*) and then monitored bioluminescence in three individually isolated recipient mice that were separated from donor animals by 15.2 cm. As with the shorter-range experiment, longer-range transmission with ten donor animals was efficient (Table 1) and included the four previously discovered categories of primary infection phenotypes (Table 2, Figure 10), albeit with nasopharyngeal initiated infection occurring at a higher frequency. Additional experiments would be needed to define precisely the contributions of separation distance and inoculation dose on the initial site of infection after airborne transmission. For airborne transmission across both 7.6- and 15.2-cm distances, transmission was on the average significantly quicker (*p* value = 0.001) for animals that progressed to a respiratory disseminated infection than for those whose infection was predominantly confined to the trachea (Figure 4B). In summary, the dynamics of primary infection after airborne transmission across 15.2 cm was similar to that across 7.6 cm.

Previous studies have shown that greater numbers of donor mice increase the efficiency of airborne transmission of SeV [27], [28], presumably due to an increase in the amount of infectious virus collectively expelled from donors. To examine the effect of the number of donor mice on the efficiency of airborne transmission, in each cage we inoculated three donor mice with either 70- or 7,000-PFU rSeV-luc(M-F*) and monitored bioluminescence and seroconversion in three individually isolated naïve mice that were separated from the donors by 15.2 cm. Under these conditions, no bioluminescence or seroconversion was observed.

## Discussion

We studied the dynamics of SeV infection in individual, living mice after contact or airborne transmission. We found that increased and longer-duration infection in the lungs of donor mice, due to a 100-fold higher inoculation dose, had no apparent effect on the frequency or timing of airborne transmission or on the dynamics of infection in recipient mice. This suggests that airborne transmission is largely determined by virus growth and expulsion from the URT, similar to findings on contact transmission [21], [29]. The dynamics of infection was largely uniform after contact transmission, in most mice initiating in the URT and then spreading to the lungs approximately one day later. In contrast, infection after airborne transmission included non-productive infection, tracheal-dominant infection, and respiratory tract disseminated infection (initiating either in the nasopharynx or in the trachea). In general, the level of primary infection in a given respiratory tissue was inversely correlated with the level of reinfection in the same tissue. Mice having a primary infection that was predominantly tracheal were relatively susceptible to reinfection, suffering greater weight loss than animals that had a primary infection disseminated throughout the respiratory tract. Overall, the data suggest that the mode of transmission determines the tropism and magnitude of primary infection, which in turn influences the tropism and magnitude of reinfection.

The transmission of respiratory viruses can occur through four routes: a) direct contact with infectious secretions, b) indirect contact with contaminated fomites, c) short-range large droplet airborne spread, and d) small droplet nuclei aerosolization. The relative contribution of each mode of transmission for a particular respiratory virus has been a topic of debate. For influenza virus, transmission in temperate climates appears to occur more frequently through large droplet or small droplet nuclei [31]–[34]. This does not appear to be the case in tropical regions as virus is unstable in aerosols at higher temperature and humidity [35]. In these regions it is hypothesized that contact transmission is the dominant mode. For human rhinoviruses the precise route of transmission remains controversial [36]–[41]. Transmission of HRSV is thought to occur through direct or indirect contact with contaminated secretions and large-particle droplets [14], [15], [42]. It is generally believed that HPIV transmission occurs via a similar route [4], [16], [43], [44], although there is little experimental evidence to support this notion [17], [19], [45]. Here we demonstrate that a parainfluenza virus can transmit by contact and through the air over short distances, presumably by large droplets. Our results support the limited clinical and experimental observations with HRSV and the HPIVs suggesting these viruses transmit predominantly by contact but also by large droplets over short distances.

Parker et al. observed 100% seroconversion of mice in direct contact with infected donor mice but inefficient or no seroconversion when naïve and donor mice were separated by 20.32 cm [27]. In contrast, van der Veen et al. found that contact transmission occurred at a rate of approximately 50–60%, short range (2.5–10.5 cm) airborne transmission occurred at a rate of 15–22%, and long distance (1.5–1.8 meters) aerosol transmission occurred at a rate of 7–32% [28], [29]. In the present study, we observed efficient airborne transmission at distances up to 15.2 cm in cages containing ten donor mice. Discrepancies between the studies may be attributed to multiple variables including differences in cage set-up, number of infected donor mice, timing of cohousing, airflow rates, and climate control. Regardless of the route of transmission, van der Veen et al. observed increased rates of transmission at higher (60–70%) over lower (40–45%) relative humidity. In the present study, relative humidity ranged from 48–85%, largely remaining between 60 and 77% (Table S1). Given that the average measured relative humidity in the present study was comparable to the humidity levels that resulted in more efficient transmission in the van der Veen study, we speculate that the difference in transmission rates between the two studies is not a function of relative humidity alone. A major finding reported here is that the dynamics of infection and protection from reinfection after short-range airborne transmission is highly diverse. A large proportion of infections initiated in the trachea, which has been previously shown to support high levels of infection [21], [30], [46]. HPIV3 infection of cotton rats leads to laryngotracheitis [47]. In human tracheobronchial epithelial cells, HPIV1 grows more efficiently than HPIV2 and HPIV3 [48]. Taken together, it is not surprising that HPIV1 is the dominant etiologic agent in outbreaks of laryngotracheobronchitis or pediatric croup throughout the world [4], [5], [17].

The mode of transmission may determine the dynamics of primary infection by dictating the site of initiation in recipient animals. Contact transmission requires the touching of mucous membranes, such as the nose or eyes, with infectious secretions [13], [49]. It follows then that direct contact between infected donor mice with high viral titers in the nasal turbinates and naïve recipient mice would more likely result in transmission of the virus to the nasal tissue of the naïve animals as opposed to deeper in the respiratory tract. Transmission of a virus through an airborne route requires an expiratory event such as coughing, sneezing, talking, or normal breathing [50]–[52]. Studies have shown these expiratory events can produce large (150 µm), intermediate (5–50 µm), and small (<5 µm) virus-containing particles [53]–[55]. Smaller-sized particles are capable of penetrating deeper into the respiratory tract [56]. Differences in the dynamics of primary infection after contact and airborne transmission described in the present study may simply be a function of the route of transmission and size of infectious particle. It is also possible that the high rate of tracheal infections observed after short-range airborne transmission are the result of particle impaction at the trachea due to the horizontal anatomy of the murine upper respiratory tract. It should be noted that in transmission experiments between intermingling mice, transmission could also occur through a short-range airborne route in addition to direct or indirect contact route.

Infection with the human paramyxoviruses HRSV, HMPV, and the HPIVs can occur throughout life [16], [57]; however unlike primary infection in the very young, subsequent infections are often milder or subclinical [16]. The mechanism behind the ability of these viruses to reinfect has been attributed to the incomplete and waning immunity that develops after primary infection with specific emphasis being placed on the serum neutralizing antibody and mucosal IgA levels [57]–[61]. One potential factor influencing the magnitude, tropism, and clinical impact of reinfection may be the mode of transmission and dynamics of primary infection, as described here for SeV transmission in mice. Thus, we hypothesize that the mode of primary infection of respiratory paramyxoviruses may also influence the severity of reinfection in other species including humans. Based on the seasonal nature of HPIV infections, in the future it will be important to address the role of temperature and humidity on the transmissibility of parainfluenza viruses through an airborne route. Bioluminescence imaging of SeV infection in living mice, the natural host, has revealed several unique phenotypes of primary infection that, in turn, influence protection from reinfection. Future studies will be aimed at understanding HPIV infection and transmission in a guinea pig model because a more detailed understanding of how these viruses transmit can have broad public health implications.

## Supporting Information

Figure S1 Definitions of regions of interest for bioluminescence data analysis. Bioluminescence curves are shown for primary infection after airborne transmission in representative animals that had (A) tracheal dominant, (B) nasopharyngeal first, and (C) tracheal first infections. In the left column, regions of interest were drawn based on correlations between external and internal anatomy as described in the Materials and Methods. In the middle column, the line of demarcation between the nasopharynx and trachea was shift up. In the right column, the line of demarcation between the trachea and lungs was shifted down. Shifting of the regions of interest did not substantial change the calculated bioluminescence phenotypes. (D) Images of regions of interest during the peak day of infection that are shown in panels A–C.
(TIF)

Table S1 Temperature and Relative Humidity for airborne transmission experiments.
(PDF)
