# Supplementary figures and images for: Mode of Parainfluenza Virus Transmission Determines the Dynamics of Primary Infection and Protection from Reinfection
Source: PLoS Pathog. 2013 Nov 21;9(11):e1003786. doi: 10.1371/journal.ppat.1003786 (PMC3836739; doi:10.1371/journal.ppat.1003786)

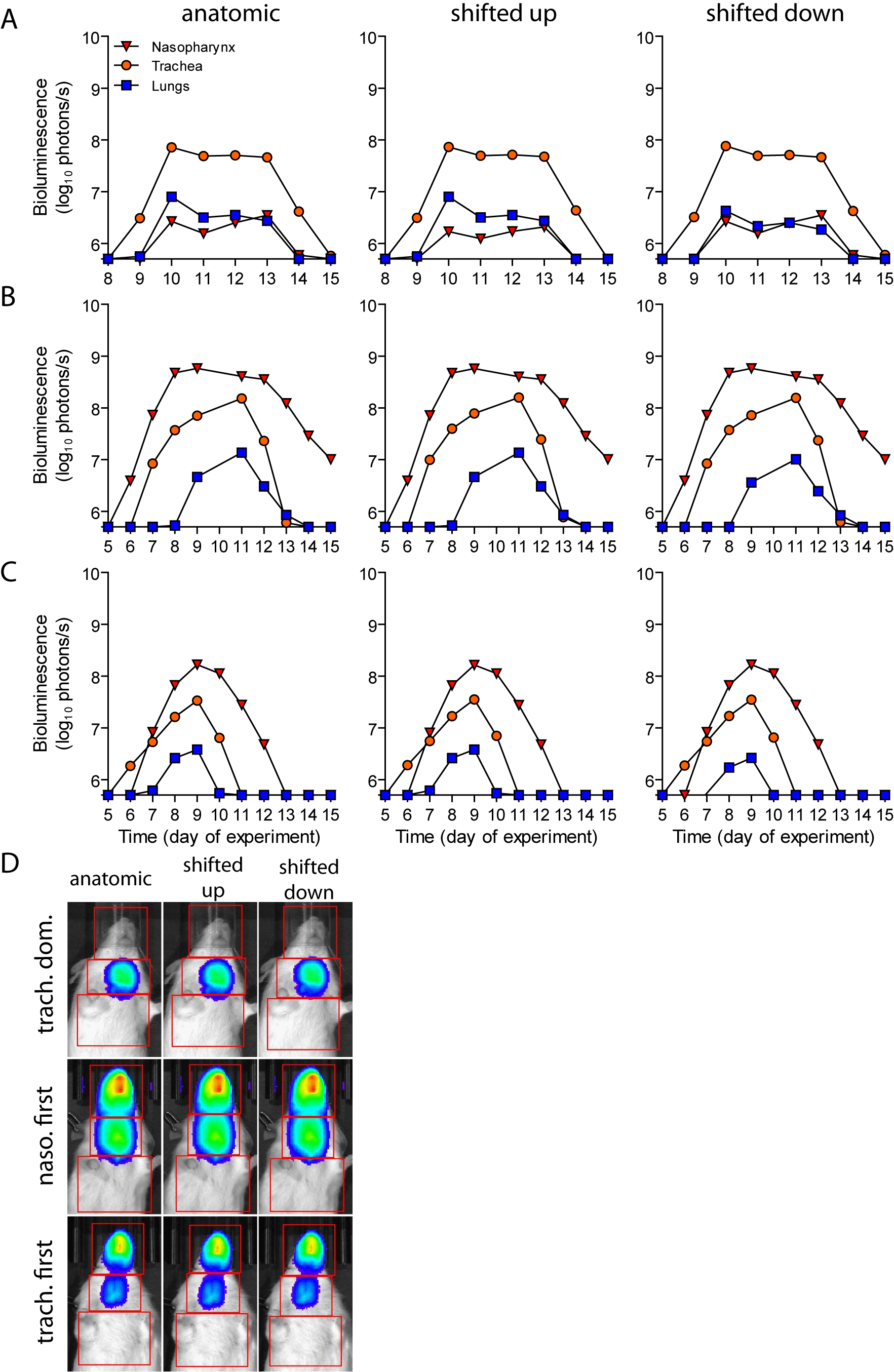

Supplement: Figure S1 — Definitions of regions of interest for bioluminescence data analysis. Bioluminescence curves are shown for primary infection after airborne transmission in representative animals that had (A) tracheal dominant, (B) nasopharyngeal first, and (C) tracheal first infections. In the left column, regions of interest were drawn based on correlations between external and internal anatomy as described in the Materials and Methods. In the middle column, the line of demarcation between the nasopharynx and trachea was shift up. In the right column, the line of demarcation between the trachea and lungs was shifted down. Shifting of the regions of interest did not substantial change the calculated bioluminescence phenotypes. (D) Images of regions of interest during the peak day of infection that are shown in panels A–C. (TIF) [file ppat.1003786.s001.tif]
