# Supplementary material for: Mode of Parainfluenza Virus Transmission Determines the Dynamics of Primary Infection and Protection from Reinfection
Source: PLoS Pathog. 2013 Nov 21;9(11):e1003786. doi: 10.1371/journal.ppat.1003786 (PMC3836739; doi:10.1371/journal.ppat.1003786)
Supplement: Table S1 — Temperature and Relative Humidity for airborne transmission experiments. (PDF) [file ppat.1003786.s002.pdf]

SUPPLEMENTAL TABLE 1: Temperature and Relative Humidity

	70 PFU cage		7000 PFU cage	
**Experiment**[a]	**Temperature**[b]	**Relative Humidity**[c]	**Temperature**	**Relative Humidity**
I	22.15 ± 0.16 (21.28-22.72)	69.63 ± 3.91 (51.55-77.97)	21.99 ± 0.25 (21.38-23.00)	67.02 ± 6.85 (48.31-79.75)
II	22.80 ± 0.22 (22.14-23.48)	72.93 ± 3.18 (65.65-81.76)	22.57 ± 0.38 (21.76-23.48)	61.92 ± 3.94 (56.24-76.87)
III	22.21 ± 0.24 (21.28-22.81)	76.50 ± 2.82 (68.70-83.80)	22.01 ± 0.21 (21.19-22.43)	81.58 ± 2.73 (66.70-85.67)
IV	ND[d]	ND[d]	22.32 ± 0.12 (21.86-22.62)	81.85 ± 5.35 (69.46-92.10)
V	ND[d]	ND[d]	22.08 ± 0.16 (21.66-22.53)	80.38 ± 4.34 (69.14-88.48)

[a]Data points measured every 30 minutes using a Hobo Data Logger for duration of the experiment. For individual experiments the following number of data points were collected : I (*n*= 620), II (*n*=661), III (*n*=714) IV (n=672), V (n=672)

[b]Temperature reported as average degrees Celsius ± SD (range)

[c]Relative Humidity reported as average % ± SD (range)

[d]Not done
